# Uniaxial Compressive Stress–Strain Relation of Recycled Coarse Aggregate Concrete with Different Carbonation Depths

**DOI:** 10.3390/ma15155429

**Published:** 2022-08-07

**Authors:** Kun Tu, Jin Wu, Yiyuan Wang, Huachao Deng, Rui Zhang

**Affiliations:** 1 Department of Civil and Airport Engineering, Nanjing University of Aeronautics and Astronautics, 29 Yudao Street, Nanjing 210016, China; 2 Jiangsu Airport Infrastructure Safety Engineering Research Center, 29 Yudao Street, Nanjing 210016, China; 3 School of Kangda, Nanjing Medical University, 88 Chunhui Road, Lianyungang 222000, China

**Keywords:** recycled coarse aggregate concrete, carbonation, uniaxial compressive loading, stress–strain curves, fitting analysis

## Abstract

**Highlights:**

- Uniaxial compressive stress–strain curves of recycled aggregate concrete (RAC) with different carbonation depth were investigated.
- The effect of carbonation depth on peak stress, strain, elastic modulus, and the relative toughness of RAC was studied.
- Stress–strain models of recycled aggregate concrete with different carbonation depths were established.

**Abstract:**

The stress–strain relation of recycled aggregate concrete (RAC) after carbonation is very important to the assessment of the durability of RAC. The objective of this study is to investigate the uniaxial compressive stress–strain curves of RAC after carbonation. In this study, the specimens were prepared with 70-mm diameter and 140-mm height cylinders, and the carbonation of the specimens was accelerated after curing 28 days. Then a uniaxial compressive loading test on the specimens was performed by using a mechanical testing machine. The results show that the peak stress ($\sigma_{0}$) and elastic modulus ($E_{c}$) of all specimens increase with the increase of carbonation depth. The ratio of ultimate strain to peak strain ($\varepsilon_{u}/\varepsilon_{0}$) and relative toughness of the specimens decrease with the increase of carbonation depth. Furthermore, carbonation has a stronger effect on natural coarse aggregate concrete (NAC) than the 50% replacement rate of RAC with similar compressive strength. Stress–strain models of recycled aggregate concrete with different carbonation depths were established according to experimental results.

## 1. Introduction

With the development of the construction industry has come a myriad of construction waste and great damage to the environment [1]. In China, 2.36 billion tons of construction waste was generated annually in the most recent decade [2]. Consequently, it is urgent to use recycled construction and demolition materials. The use of recycled aggregate (RA) in concrete opens a whole new range of possibilities for reusing materials in construction. Reuse of waste concrete as RA in new concrete is beneficial from the viewpoint of environmental protection and preservation of resources, as it reduces the use of natural materials used in concrete production [3]. It presents a more environmentally friendly alternative destination for this waste [4]. Using recycled aggregate could save about 60% of limestone resources and reduce CO_2_ emissions by about 15–20% [5]. However, the strength of RAC decreases with the increase of the replacement percentage of recycled aggregate due to the presence of micro-cracks and old cement paste that has adhered to the original aggregates [6,7,8]. Azevedo pointed out that the substitution of more than 25% of construction and demolition waste (CDW) for sand requires relatively larger amounts of water in the mortar, and the water absorption of the mortar increased with the levels of incorporated CDW [9]. Because of the high variability in the characteristics of recycled aggregates, in order to obtain better properties, a deeper classification of the constituents should be carried out [10]. The premix process can fill up some pores and cracks, resulting in a denser concrete, an improved interfacial zone around recycled aggregate, and thus a higher strength when compared with the traditional mixing approach [11].

In addition, many researchers have argued that the carbonation resistance of RAC was worse than that of natural aggregate concrete (NAC) [12,13,14], which may lead to the corrosion of reinforcement, causing safety hazards and great economic losses [15]. There are many factors affecting the carbonation of RAC, such as the cement matrix, aggregate, mixing method, external load, and external environment, etc. [14]. Consequently, it is necessary to strengthen recycled coarse aggregate (RCA) or recycled aggregate concrete (RAC) itself in order to obtain RAC with better properties.

Some researchers found that the properties of RAC could be improved by adding pozzolan, silica fume, and rubber particles and fibers into RAC [16,17,18,19,20]. Other researchers found that the properties of RAC can be improved by carbonation which includes carbon conditioning and carbon curing. Carbon conditioning is the injection of CO_2_ into recycled aggregate, accomplished with the assistance of a sealable carbonation chamber [21]. Zhang et al. [22] and Zhan et al. [23] found carbonation can improve RCA in physical and mechanical properties. The apparent density of RCA was significantly increased, whereas the water absorption and crushing value of RCA were significantly decreased after carbonation. Carbon curing relies upon carbonation reaction between CO_2_ and cement paste, and it carbonates entire concrete blocks after concrete mixing [18]. Zhan et al. [24] found that carbon curing improved rapidly the compressive strength of RAC with strength gains ranging from 108% to 151% higher than conventional moisture curing. Zhan et al. [25] and Xuan et al. [26] found that carbon-curing conditions will lead to improvements of RAC strength.

The widespread adoption of RAC requires not only a better understanding of its mechanical properties and durability but also the availability of guidelines on designing reliable RAC structures [27]. In order to guide the design of RAC structures, the stress–strain relationship of RAC has been preliminarily investigated [28,29,30,31]. In addition, Luo et al. [32] investigated stress–strain curves of fully carbonated RAC and found that carbonated RAC improved the compressive strength and elastic modulus of RAC. However, less information is available on the effect of carbonation depth on the stress–strain relation of RAC.

The objective of this study is to evaluate the peak stress, elastic modulus, strain, relative toughness and establish the stress–strain model of RAC with different carbonation depths according to experimental results.

## 2. Experimental Method

### 2.1. Materials and Mixture Proportions

**Figure 1:**
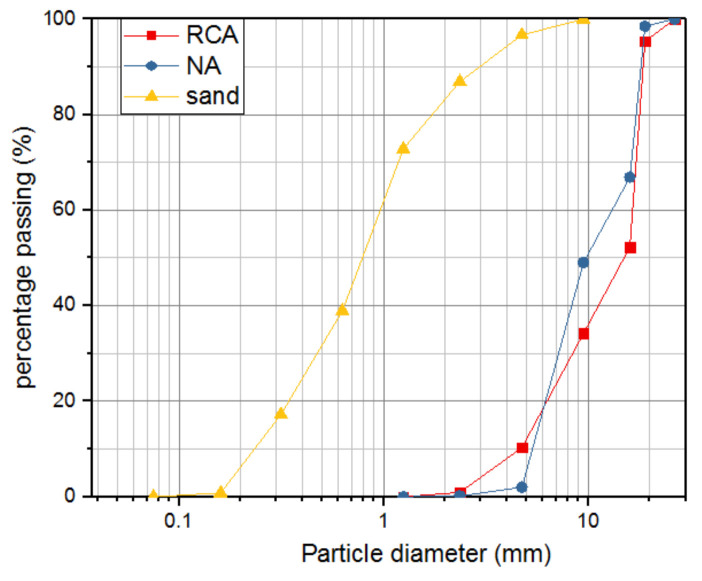
Grading curve of aggregates.

Ordinary Portland cement of grade 42.5 was used in this study. The cement properties are shown in Table 1, and river sand with particle size less than 4.75 mm was used as fine aggregates. NCA was from gravel, and RCA was produced from Nanjing Shoujia Renewable Resources Utilization Company (Nanjing, China). After the concrete residues were transported to the company, a jaw crusher was employed in order to reduce the size of the large pieces of concrete residues. After being crushed, the residues were cleaned and graded ready for the planned tests. The crushing values of NCA and RCA were measured according to Pebbie and crushed stone for building (GB/T14685-2011) in China. The size grading of RCA and NCA were similar to ranging from 5 mm to 20 mm, and satisfied the standard for technical requirements and test method of sand and crushed stone (or gravel) for ordinary concrete (JGJ52-2006) in China. Figure 1 shows the sieving results of aggregates, and the properties of NCA and RCA are shown in Table 2.

Due to the high water absorption of RCA, pre-soaking was used to make RCA reach the saturated surface dry (SSD) condition. The mix design proportions of RAC and NAC are listed in Table 3. Note that the replacement ratio of specimens is calculated by volume. The water–cement ratio and sand ratio were adjusted to obtain similar 28-day compressive strengths of NAC and RAC based on the technical code on the application of recycled concrete (DG/TJ08-2018) in China.

The specimens with 70-mm diameter and 140-mm height cylinders were cast in a PVC mould. After curing for 28 days, the compressive strengths of RAC cubes (100 mm × 100 mm × 100 mm) with replacement ratios of 0%, 50% and 100% were 37.5 MPa, 36.4 MPa and 31.1 MPa, respectively.

### 2.2. Accelerated Carbonation Procedure

The acceleration of concrete carbonation was performed in a carbonation experiment chamber. The temperature was 20 °C, the humidity was 60% and the CO_2_ concentration was 20%. All specimens were wax-sealed at both ends to conduct side-only carbonation. To achieve different carbonation depths, carbonation periods were set to 0, 14, 28, 42, 56, 70, and 84 days.

The carbonation depth is a quantity index that characterizes the degree of carbonation. A 1/3-length cylinder was split when measuring carbonation depth, and the cross-section of the remaining piece was sealed with wax for further carbonation and measurement. For the piece after splitting, drop 1% phenolphthalein alcohol solution (alcohol solution contains 20% distilled water) on the cross-section. Then a measurement point was marked every 45° on the circular section and the depth of carbonation was measured at each measurement point. The average value of carbonation depth was taken as the carbonation depth of the specimens,

(1) $$\bar{d_{t}}=\frac{1}{n}\sum_{1}^{n}d_{i}$$

where *d_t_* is the average carbonation depth (mm) after carbonation *t* (*d*) of the test, *d_i_* is the carbonation depth of measuring point (mm), and *n* is the total number of measuring points.

### 2.3. Uniaxial Compressive Loading Test

**Figure 2:**
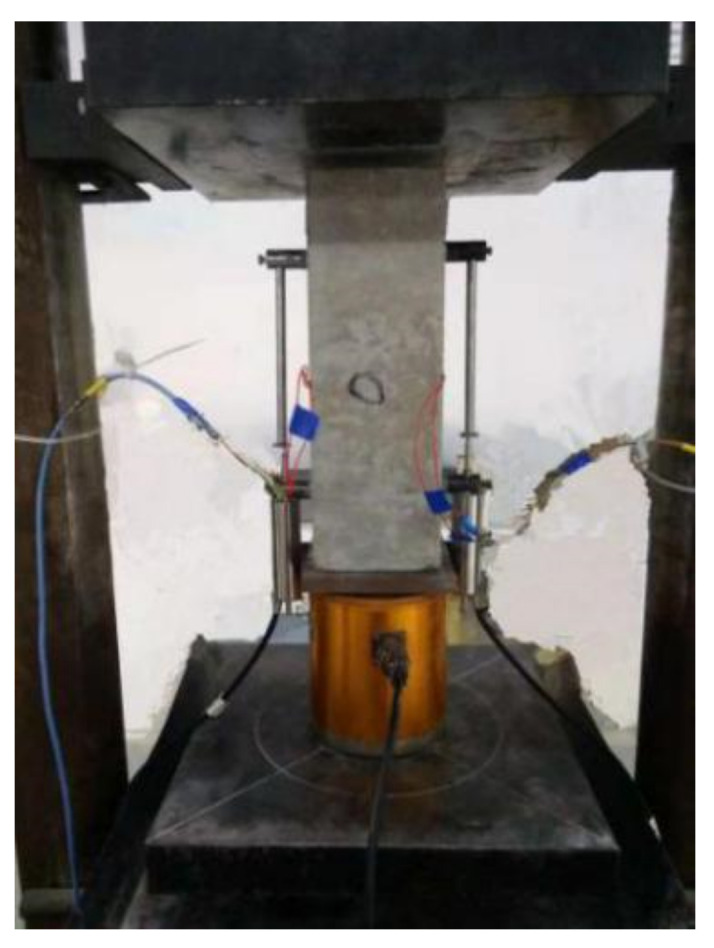
Test setup of loading and measurement.

A uniaxial compressive loading test was performed by using a mechanical testing machine. Two displacement meters were fixed on the middle part of both sides of the specimen, and the displacement in the middle part of the specimen (170–180 mm) was measured in this test as shown in Figure 2. A load sensor was placed under the steel pads. The dynamic data acquisition system was used to collect the test data.

The peak strain ($\varepsilon_{0}$) is the strain corresponding to peak stress and the ultimate strain ($\varepsilon_{u}$) is the strain corresponding to the stress which is 50% of peak stress at the descending part of the stress–strain curve.

The elastic modulus (*E_c_*) was calculated with the following expression [33,34]:

(2) $$E_{c}=\frac{\sigma_{0.4}-\sigma_{0}}{\varepsilon_{0.4}-\varepsilon_{0}},$$

where $\sigma_{0}$ is peak stress and $\sigma_{0.4}$ is the stress corresponding to 40% of the peak stress, $\varepsilon_{0}$ is the peak strain, and $\varepsilon_{0.4}$ is the corresponding to $\sigma_{0.4}$.

## 3. Experimental Results and Discussion

### 3.1. Carbonation Depth

**Figure 3:**
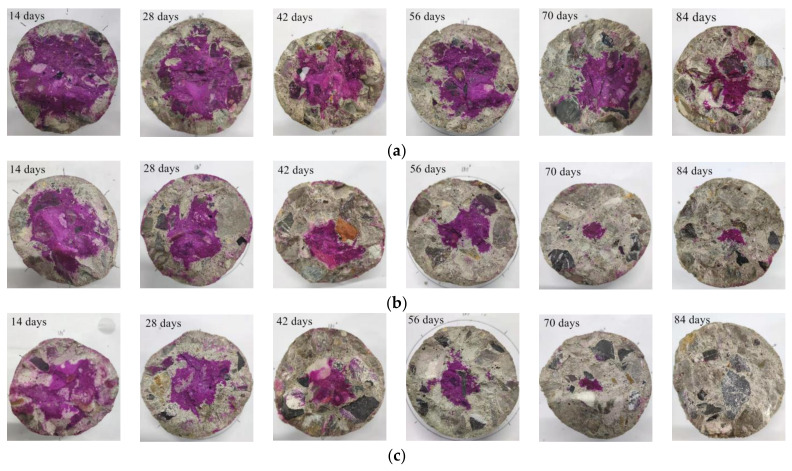
Typical specimens after different carbonation periods. (**a**) NAC; (**b**) RAC50; (**c**) RAC100.

**Figure 4:**
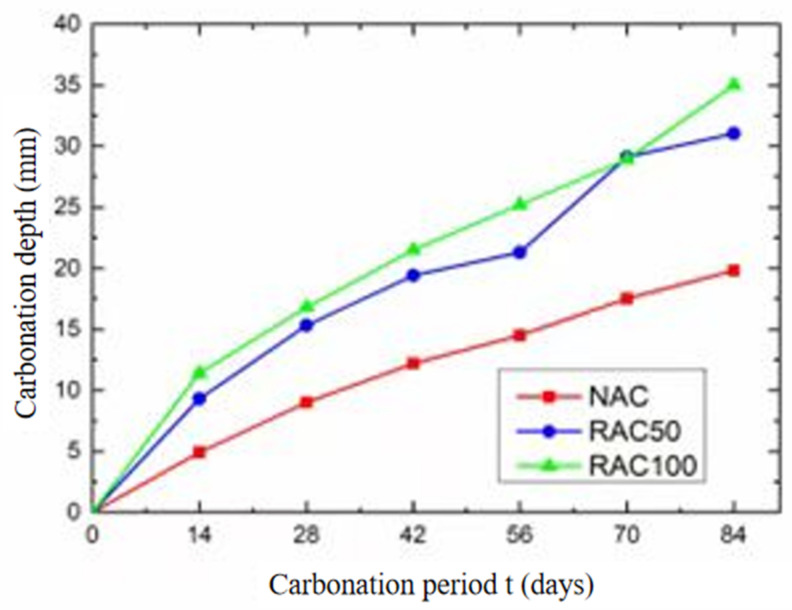
Carbonation depth versus carbonation periods.

Figure 3 shows carbonated specimens with 0%, 50%, and 100% RCA replacement ratios. The correlation between carbonation depths and carbonation periods is shown in Figure 4. It can be seen that the carbonation depth of specimens increased as the replacement ratio of RCA increased due to the increase of the porosity of RAC. This agreed with the findings observed by C. Thomas and Miguel Bravo [35,36].

Moreover, the RAC100 was fully carbonated at 35.0 mm of carbonation depth, whereas RAC50 and NAC were carbonated at 31.0 and 19.2 mm, respectively. Notably, the carbonation depth of RAC50 was 12% higher than that of NAC under a similar 28-day compressive strength. However, the carbonation resistance of RAC can be improved upon by adding other materials, such as fly ash [14], etc. Consequently, it is possible to apply RAC in field applications.

### 3.2. Failure Pattern

**Figure 5:**
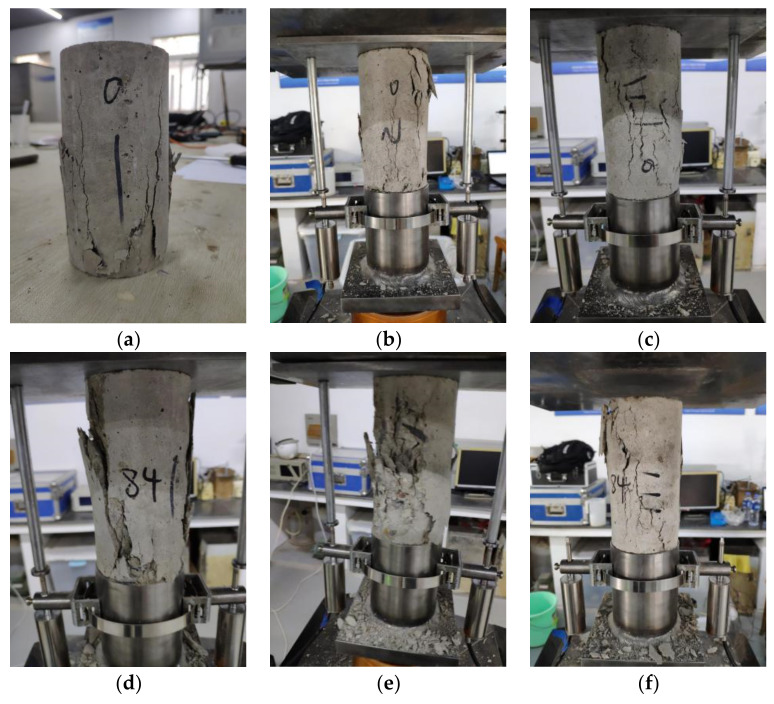
The failure pattern of RAC and NAC specimens. (**a**) NAC-0.0 mm; (**b**) RAC50-0.0 mm; (**c**) RAC100-0.0 mm; (**d**) NAC-19.8 mm; (**e**) RAC50-31.0 mm; and (**f**) RAC100-35.0 mm.

**Figure 6:**
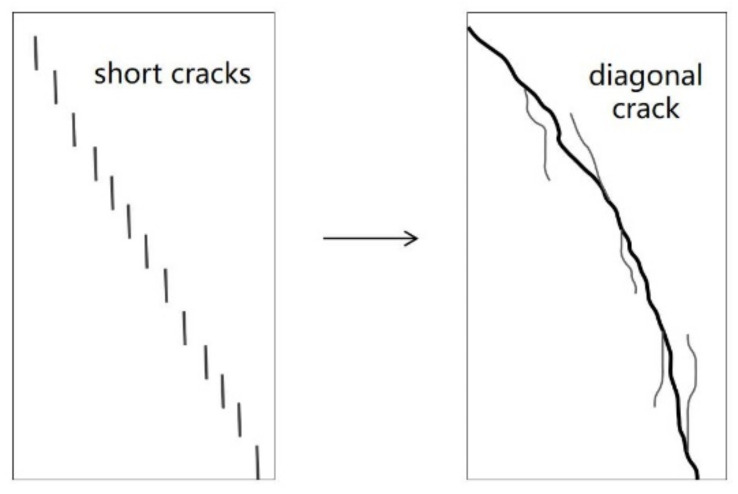
The development of the failure of carbonated RAC specimens.

Failure patterns of RAC and NAC specimens with different carbonation depths are compressive failure modes, as shown in Figure 5. For non-carbonated RAC and NAC specimens, there were a few cracks that were parallel to the loading direction, and the surfaces were slightly spalling. There is no significant difference in failure patterns of uncarbonated RAC and NAC specimens, as shown in Figure 5a–c. In contrast, for highly carbonated RAC50 and RAC100 specimens, as shown in Figure 5e,f, lots of short cracks were parallel to the loading direction, but these short cracks formed one or several long cracks. Finally, there were diagonal cracks that passed through the whole specimens, as shown in Figure 6. However, carbonated NAC specimens had the same failure pattern with uncarbonated NAC, and the spalling happened in carbonated NAC, as shown in Figure 5d. Therefore, the effect of carbonation on the failure pattern of RAC is more obvious than that of NAC. This is explained as follows: for carbonated RAC, carbonation did not change the pore and microcrack in RCA, but there were obvious micro-cracks on the interface between RCA and the new cement matrix, and the structure was looser in the interface transition zone (ITZ). Yang et al. [37] found the above phenomenon by comparing scanning electron micro-graphs of carbonated RAC with that of carbonated NAC. Consequently, carbonation mainly changes the microstructure of the paste and interface transition zone. Short cracks occurred in RAC with the increase of loading, but the more pore and microcracks in RCA prevented the development of short cracks. Therefore, lots of short cracks occurred and formed one or several long diagonal cracks, resulting in the diagonal band failure of specimens.

In Figure 5f, the failure mode of RAC100-35.0 mm, which was fully carbonated, is similar to the findings in the literature [38]. Although the interior of the fully carbonated concrete has a high degree of compactness, a large number of original cracks inside RCA was responsible for the failure of carbonated RAC.

### 3.3. Uniaxial Compressive Stress–Strain Curves

**Figure 7:**
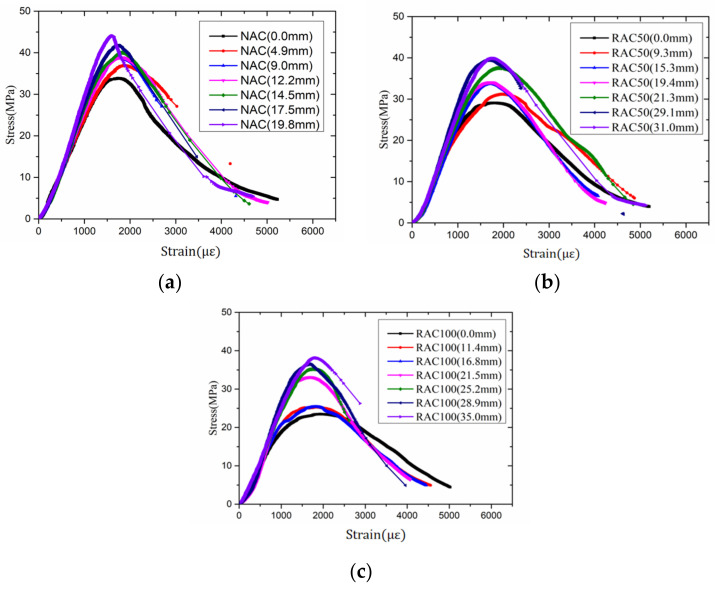
Uniaxial stress–strain curves of the specimens with different carbonation depth. (**a**) NAC; (**b**) RAC50; and (**c**) RAC100.

The uniaxial compressive stress–strain curves of the specimens with different carbonation depths are shown in Figure 7. It is shown that the stress–strain curves of the specimens exhibit similar features, whereas the replacement rate of RCA and carbonation depth showed an obvious impact on the stress–strain relation of RAC.

The peak stress and slope of the ascending part of the curve declined with the increase of the replacement rate of RCA, which conformed to findings from other researchers [29,38]. Furthermore, as carbonation depths increased, peak stress increased and both the ascending and descending part of the stress–strain curves became steeper. During a uniaxial compressive loading experiment, the highly carbonated specimens were more brittle.

#### 3.3.1. Peak Stress

**Figure 8:**
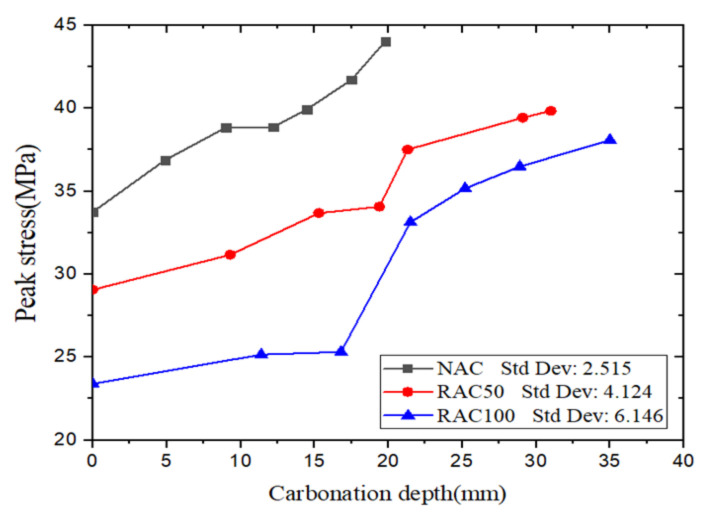
Peak stress versus carbonation depth.

Figure 8 shows the relationship between peak stress and carbonation depth. The results show that the peak stress of NAC, RAC50, and RAC100 specimens increased with the increase of carbonation depth. This is caused by the reactions between CO_2_ and hydration products of cement, such as Ca(OH)_2_ and C-S-H, which can reduce the porosity of concrete [33,34,37,38,39]. The peak stress of the concrete was increased by reducing the porosity [40,41,42].

When the carbonation depth reached about 20 mm, the peak stress of NAC, RAC50, and RAC100 specimens increased by about 30.5%, 17.6%, and 41.6%, respectively, compared with uncarbonated specimens. The result shows that the peak stress of NAC increased much more than that of RAC50 with the same carbonation depths. Moreover, the peak stress of fully carbonated RAC100 increased by about 62.8%; hence carbonation can increase the strength of RAC, which is consistent with findings by other researchers [24,25,26]. In Figure 8, it is found that the standard deviation values of the peak stress of RAC100 is higher than that of RAC50 and NAC, the peak stress has a clear jump from 16.8 to 21.5 mm of carbonation of RAC100. The reason may be due to the discreteness of RAC. Further experiments need to be performed in order to investigate the phenomenon.

#### 3.3.2. Strain

**Figure 9:**
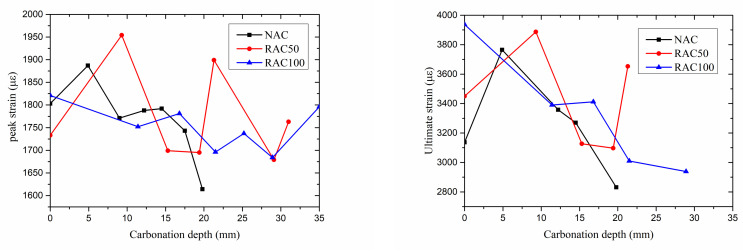
Peak strain and ultimate strain versus carbonation depth.

The peak strain and ultimate strain of the specimens with different carbonation depths are shown in Figure 9. It shows that carbonation depth has no significant effect on the peak strain of all specimens. However, the ultimate strain of NAC and RAC100 decreased with the increase of carbonation depth.

**Figure 10:**
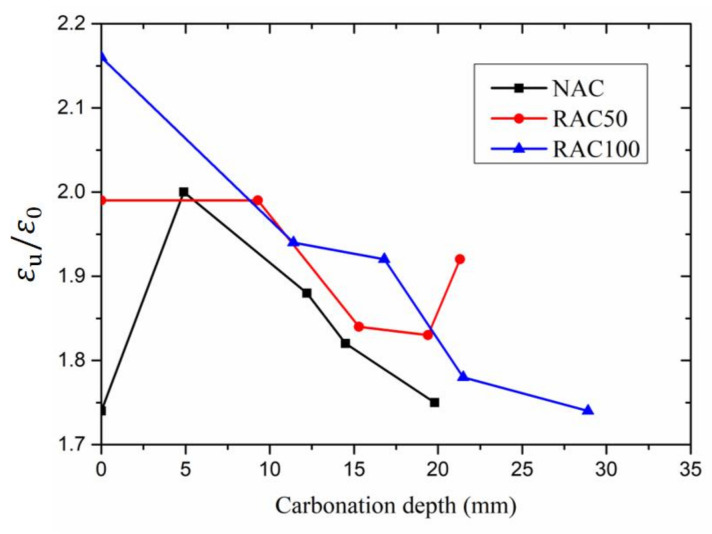
Ratio of ultimate strain to peak strain versus carbonation depth.

The ratio of the ultimate strain to peak strain ($\varepsilon_{u}/\varepsilon_{0}$) describes the trend of the descending part of stress–strain curves [43]. Figure 10 shows the relationship between the ratio of ultimate strain to peak strain and carbonation depth. The results show that the ratio of ultimate strain to peak strain of RAC50 is roughly larger than that of NAC. This means the descending part of the stress–strain curve of RAC50 is flatter than that of NAC, and the results match with the descending part of stress–strain curves. Additionally, the ratios of ultimate strain to peak strain of NAC, RAC50, and RAC100 specimens decreased with the increase of carbonation depth. This means the descending part of the stress–strain curves of these specimens becomes steeper and steeper due to the carbonation. This is consistent with the rapid destruction of specimens after carbonation.

#### 3.3.3. Elastic Modulus

**Figure 11:**
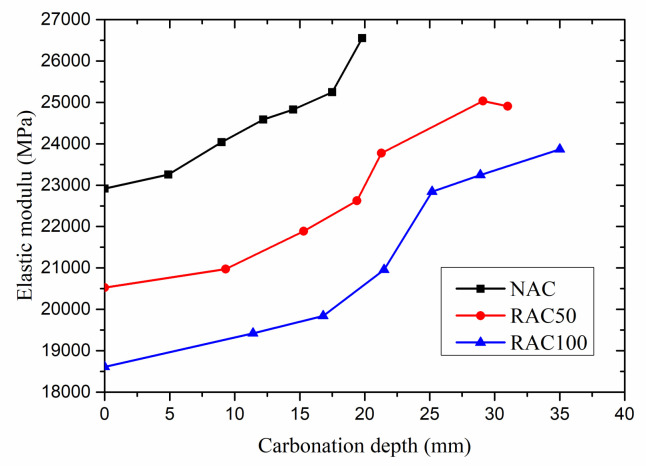
Elastic modulus versus carbonation depth.

Figure 11 shows the relationship between the elastic modulus and carbonation depth. It can be observed that the elastic modulus of RAC50 is significantly lower than that of NAC. The reasons are as follows. On the one hand, the decrease in the elastic modulus of RAC50 was attributed to the weak and porous recycled coarse aggregates [5,44]. On the other hand, the elastic modulus may decrease due to the cracks between old mortar and aggregates and the micro-cracks which occur in the process of the production of RCA [45].

Moreover, the elastic modulus of NAC, RAC50, and RAC100 specimens increased with the increase of carbonation depth. Oğuzhan Çopuroğlu [46] and Han Jian De [47] obtained similar results by using nanoindentation technology. Concrete specimens become denser with the increase of carbonation depth, which is due to the carbonation of concrete. In the carbonation, the chemical reaction between CO_2_ entered in the concrete and Ca(OH)_2_ in the cement produces insoluble CaCO_3_, which deposits in the pores of concrete.

When the carbonation depth reached about 20 mm, the elastic modulus of NAC, RAC50, and RAC100 specimens increased by about 15.8%, 10.2%, and 12.6%, respectively, compared to uncarbonated specimens. The result shows that the elastic modulus of NAC is higher than that of RAC50 with the same carbonation depth. When the RAC100 is fully carbonated, its elastic modulus has increased by about 28.3%.

#### 3.3.4. Relative Toughness

The area under the stress–strain curve can generally be considered as a measure of the toughness of materials [48]. However, researchers’ definition of toughness is not universal. Wengui Li [41] and Sufen Dong [49] regarded the area under the stress–strain curve as toughness directly. Zemei Wu [50] and Jinyang Jiang [51] defined the relative toughness as the ratio of the area under the curve to that of the ascending portion before the peak stress.

**Figure 12:**
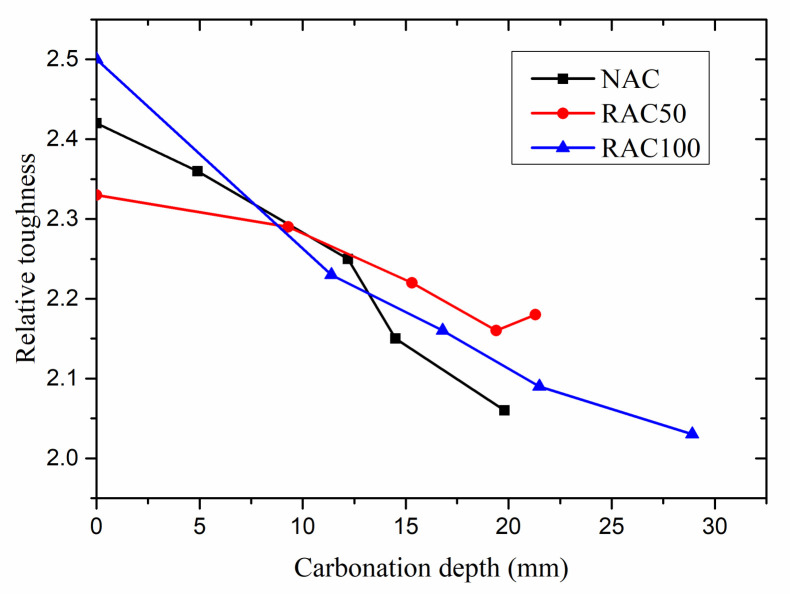
Relative toughness versus carbonation depth.

To avoid misjudgment due to much higher peak stress after carbonation, the ratio of the area under the curve before 50% of peak stress at the descending part to that of before peak stress was defined as relative toughness in this study. Figure 12 shows the relationship between relative toughness and carbonation depth. The relative toughness of uncarbonated NAC is higher than that of RAC50. This may be due to the weak and porous RCA. The relative toughness of NAC, RAC50, and RAC100 specimens decreased overall with the increase of carbonation depth. When the carbonation depth reached about 20 mm, the relative toughness of NAC, RAC50, and RAC100 specimens decreased by about 14.6%, 7.1%, and 16.2%, respectively, compared with non-carbonated specimens. The results show that the relative toughness of NAC reduced faster than that of RAC50 with the increase of carbonation depth.

## 4. Stress–Strain Relation of Carbonated RAC and NAC

Xiao [29] and Wu [31] found that the analytical equations proposed by Guo [52] and adopted by the code for design of concrete structures (GB 50010-2002) in China to describe the complete stress–strain curve of NAC were also applicable to RAC. Hence, in this study, the equations are adopted to analyze the stress–strain relation of carbonated RAC and NAC are shown as follows:

(3) $$\begin{array}{l} y=\left\{\begin{array}{l} ax+(3-2a)x_{}^{2}+(a-2)x_{}^{3},x\leq 1\\ \frac{x}{b{\left(x-1\right)}_{}^{2}+x},x\geq 1 \end{array}\right.\\ x=\frac{\varepsilon }{\varepsilon_{c}},y=\frac{\sigma }{\sigma_{c}} \end{array}$$

where *a* and *b* are parameters affecting the ascending and descending part of the curve, respectively; $\varepsilon_{0}$ is the peak strain; $\sigma_{0}$ is the peak stress.

**Figure 13:**
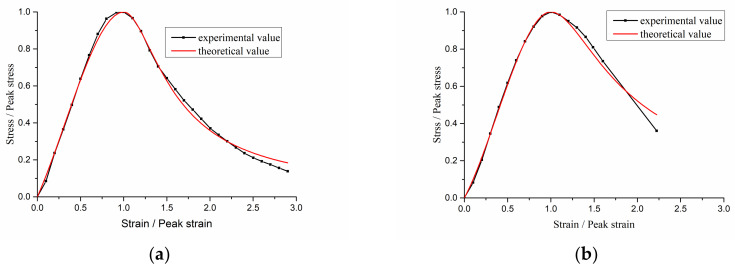

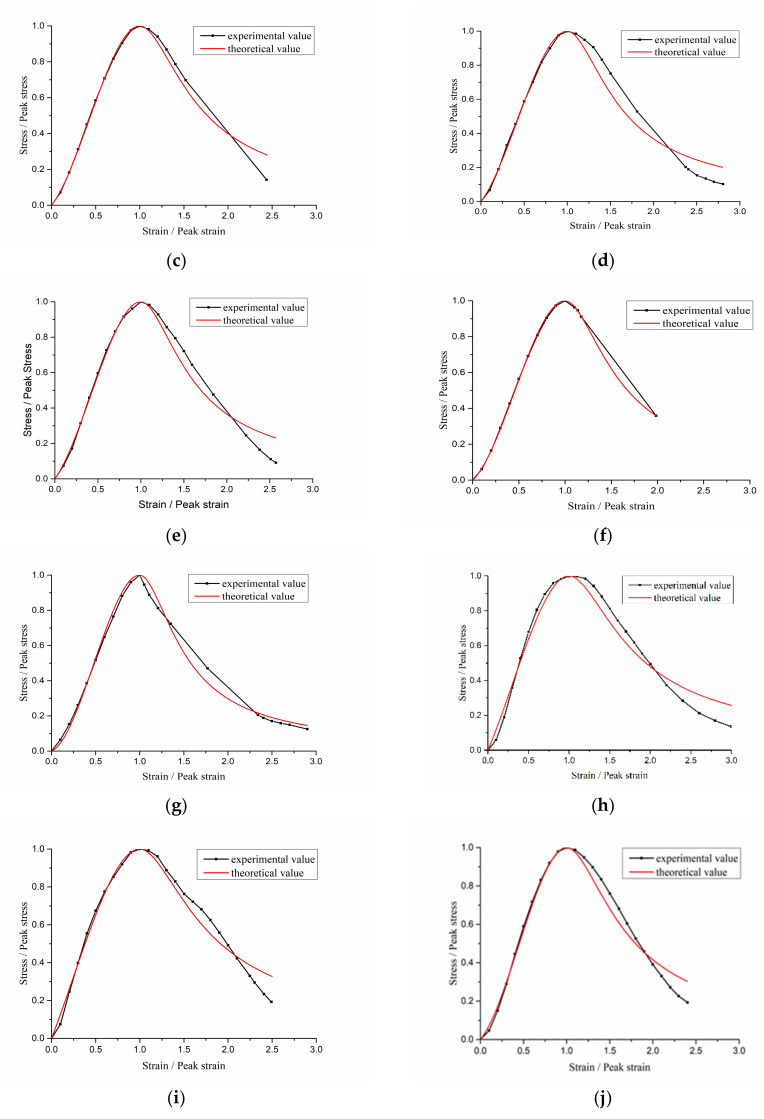

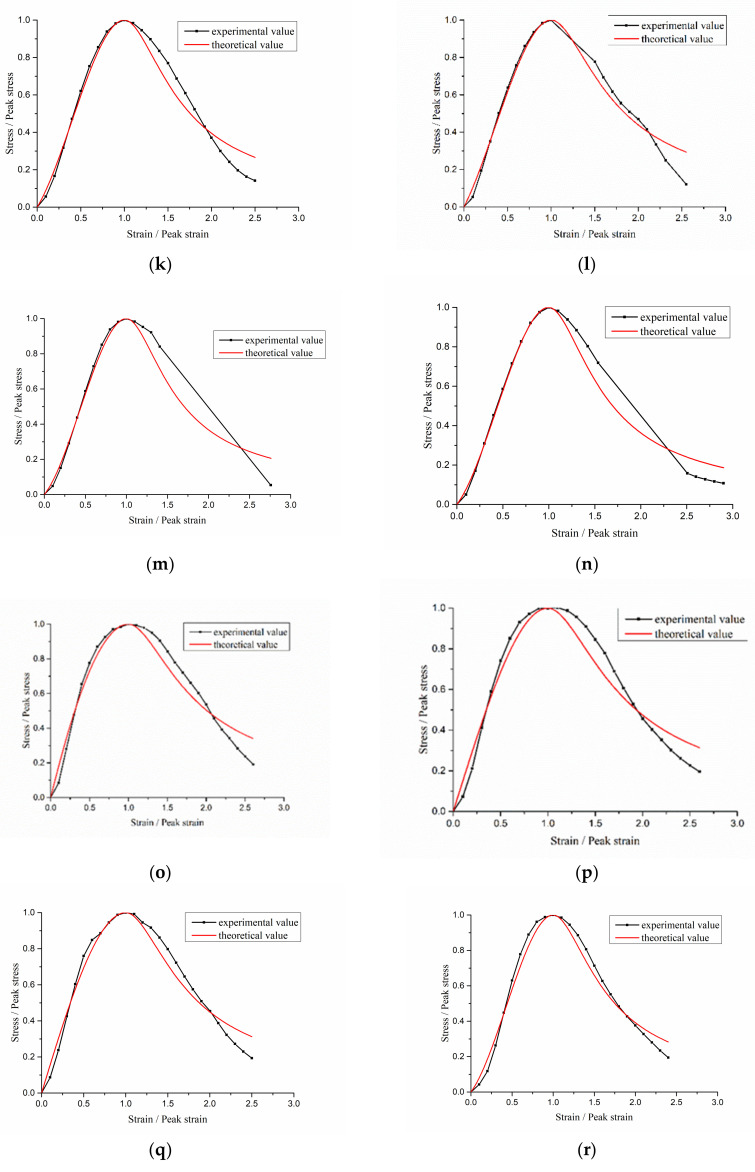

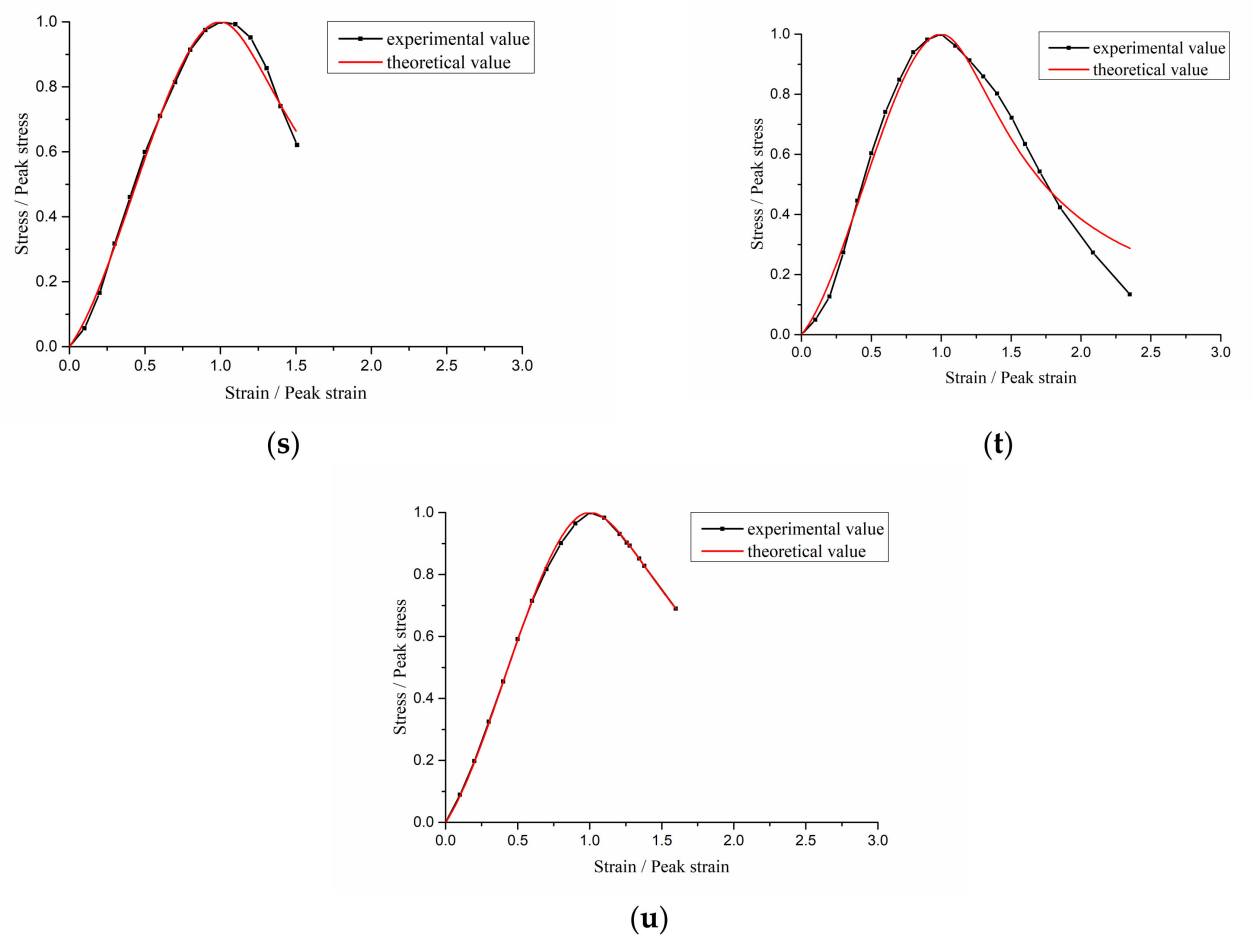
The fitting results of stress–strain relation. (**a**) NAC-0.0 mm; (**b**) NAC-4.9 mm; (**c**) NAC-9.0 mm; (**d**) NAC-12.2 mm; (**e**) NAC-14.5 mm; (**f**) NAC-17.5 mm; (**g**) NAC-19.8 mm; (**h**) RAC50-0.0 mm; (**i**) RAC50-9.3 mm; (**j**) RAC50-15.3 mm; (**k**) RAC50-19.4 mm; (**l**) RAC50-21.3 mm; (**m**) RAC50-29.1 mm; (**n**) RAC50-31.0 mm; (**o**) RAC100-0.0 mm; (**p**) RAC100-11.4 mm; (**q**) RAC100-16.8 mm; (**r**) RAC100-21.5 mm; (**s**) RAC100-25.2 mm; (**t**) RAC100-28.9 mm; and (**u**) RAC100-35.0 mm.

The First Optimization software developed by 7D-Soft High Technology Inc. was used to find the optimum parameters of the ascending part and the descending part according to the test data based on Equation (3). Parameter *a*, *b* and corresponding correlation coefficients are shown in Table 4. The fitting results of the stress–strain relation are shown in Figure 13. The results show that the test values are in good agreement with the theoretical values and correlation coefficients are over 0.9. It is proven that Equation (3) is also applicable to carbonated RAC and NAC.

Parameter *a* represents the relative slope of the ascending part of the curve, and a smaller value means a flatter ascending part [50]. Parameter *a* of uncarbonated NAC and RAC50 are similar, whereas that of RAC100 is nearly 70% higher than them. This potentially indicates that parameter *a* depends on the compressive strength more than the replacement rate of RCA. Besides, parameter *a* generally shows a downward trend with the increases in the carbonation depth. This means carbonation increased the relative flatness of the ascending part of stress–strain curves.

Parameter *b* presents the relative slope of the descending part of the curve. A bigger *b* value means a steeper descending part of the curve and therefore poorer toughness performance [51]. It can be clearly observed that parameter *b* increases with the increase of carbonation depth, meaning that carbonation increased the relative steepness of the descending part of stress–strain curves.

In addition, the correlation coefficients of NAC are higher than those of RAC50 and RAC100, which may be due to the discreteness of RAC [28].

The research results show that low strength of interfacial transition zone is the main factor of the size effect of concrete. Consequently, the size effect on the strength of recycled aggregate concrete becomes obvious because the strength of interfacial transition zone of recycled aggregate concrete is lower than that of ordinary concrete [53]. The compressive stress–strain model of recycled coarse aggregate concrete in this study is mainly used in the finite element method (FEM) analysis of concrete structure. The element size in the concrete structure analysis is small, so the size effect on strength and brittleness will be small. Therefore, the experimental results in Figure 13 will be directly applied to FEM analysis of concrete structure in the engineering practice.

## 5. Summary

In this study, the uniaxial compressive behavior of RAC with different carbonation depths was experimentally investigated, and the effects of carbonation depths on NAC and RAC were compared by experimental results. The main results are summarized as follows.

(1) During the accelerated carbonation, the values of the carbonation depth of RAC increases as the replacement ratio of recycled coarse aggregate increases. At 84 days, RAC100 was fully carbonated and the carbonation depth was 35.0 mm, whereas that of RAC50 and NAC were 31.0 and 19.2 mm, respectively.
(2) The peak stress and elastic modulus of RAC and NAC specimens increased with the increase of carbonation depth, and the peak stress of fully carbonated RAC100 increased by about 62.8% and elastic modulus has increased by about 28.3%. The ratio of ultimate strain to peak strain and relative toughness of RAC and NAC specimens decreased overall with the increase of carbonation depth. When the carbonation depth reached about 20 mm, the relative toughness of RAC50 and RAC100 specimens decreased by about 7.1% and 16.2%, respectively, compared with non-carbonated specimens.
(3) Stress–strain models of recycled aggregate concrete with different carbonation depths were established, and the experimental values are in good agreement with the theoretical values and correlation coefficients are over 0.9. Carbonation decreased the relative slope of the ascending part and increased the relative steepness of the descending part of stress–strain curves of recycled aggregate concrete.

## Figures and Tables

**Table 1:** Properties of cement.

Property	Value
Type and class	P.O 42.5
Specific surface area (m^2^ kg^−1^)	335
Initial and final setting times (min)	215/265
28-day compressive and flexural strength (MPa)	49.8/8.7
SO_3_ (%)	2.4
MgO (%)	1.8
CaO (%)	62.6
SiO_2_ (%)	21.2
Al_2_O_3_ (%)	5.6
Fe_2_O_3_ (%)	4.6

**Table 2:** Properties of the NCA and RCA.

Aggregate Type	Coarse Aggregate Grading (mm)	Apparent Density (kg/m^3^)	Water Absorption (%)	Crushing Value (%)
NCA	5–20	2773	1.08	3.2
RCA	5–20	2541	5.12	14.2

**Table 3:** Mix design proportions of NAC and RAC.

Concrete Type	Water (kg/m^3^)	Cement (kg/m^3^)	Water to Cement Ratio	NCA (kg/m^3^)	RCA (kg/m^3^)	Fine Aggregates (kg/m^3^)
NAC	195	274.6	0.71	1131.1	0.0	754.1
RAC50	200	333.3	0.60	566.8	519.0	694.2
RAC100	205	410.0	0.50	0.0	1006.6	643.5

**Table 4:** Fitting results.

Specimens	Coefficient *a*	Correlation Coefficient	Coefficient *b*	Correlation Coefficient
NAC-0.0 mm	1.057	0.998	3.568	0.993
NAC-4.9 mm	0.871	0.999	1.835	0.960
NAC-9.0 mm	0.624	0.999	3.010	0.950
NAC-12.2 mm	0.653	0.999	3.432	0.947
NAC-14.5 mm	0.674	0.999	3.466	0.944
NAC-17.5 mm	0.473	0.999	3.648	0.999
NAC-19.8 mm	0.214	0.997	4.706	0.988
RAC50-0 mm	1.099	0.990	2.170	0.943
RAC50-9.3mm	1.213	0.997	2.284	0.943
RAC50-15.3 mm	0.540	0.998	2.813	0.950
RAC50-19.4 mm	0.766	0.996	3.065	0.931
RAC50-21.3 mm	0.926	0.996	2.554	0.911
RAC50-29.1mm	0.571	0.997	3.434	0.930
RAC50-31.0 mm	0.613	0.999	3.505	0.959
RAC100-0 mm	1.841	0.986	1.962	0.921
RAC100-11.4 mm	1.482	0.985	2.220	0.926
RAC100-16.8 mm	1.551	0.988	2.445	0.945
RAC100-21.5 mm	0.636	0.986	3.104	0.972
RAC100-25.2 mm	0.636	0.999	3.032	0.957
RAC100-28.9 mm	0.558	0.995	3.193	0.944
RAC100-35.0 mm	0.705	0.999	2.005	0.999
